# Physiological and Genotypic Characteristics of Nitrous Oxide (N2O)-Emitting *Pseudomonas* Species Isolated from Dent Corn Andisol Farmland in Hokkaido, Japan

**DOI:** 10.1264/jsme2.ME15155

**Published:** 2016-04-22

**Authors:** Yanxia Nie, Li Li, Reika Isoda, Mengcen Wang, Ryusuke Hatano, Yasuyuki Hashidoko

**Affiliations:** 1 Research Faculty and Graduate School of Agriculture, Hokkaido UniversityKita 9, Nishi 9, Kita-ku, Sapporo 060–8589Japan

**Keywords:** nitrous oxide (N_2_O) emitter, *Pseudomonas* sp., *nosZ*-missing and *nosZ*-harboring denitrifiers, Andisol corn farm

## Abstract

Dent corn Andisol at the Hokkaido University Shizunai Livestock Experimental Farm actively emits nitrous oxide (N_2_O). In order to screen for culturable and active N_2_O emitters with high N_2_O emission potential, soft gel medium containing excess KNO_3_ was inoculated with soil suspensions from farm soil samples collected at different land managements. Dominant bacterial colonies were searched for among 20 of the actively N_2_O-emitting cultures from post-harvest soil and 19 from pre-tilled soil, and all isolates were subjected to the culture-based N_2_O emission assay. Ten active N_2_O-emitting bacteria, four from post-harvest soil and six from pre-tilled soil, out of 156 isolates were identified as genus *Pseudomonas* by 16S rRNA gene sequencing. These N_2_O emitters showed clear responses to NO_3_^−^ within a neutral pH range (5.5–6.7), and accelerated N_2_O production with 1.5–15 mM sucrose supplementation, suggesting the production of N_2_O during the denitrification process. However, the negative responses of 6 active N_2_O emitters, 3 from post-harvest soil and 3 from pre-tilled soil, out of the 10 isolates in the acetylene-blocking assay suggest that these 6 N_2_O emitters are incomplete denitrifiers that have lost their N_2_O reductase (N_2_OR) activity. Although the PCR assay for the denitrification-associated genes, *narG* and *nirK/S*, was positive in all 10 *Pseudomonas* isolates, those negative in the acetylene-blocking assay were *nosZ*-negative. Therefore, these results imply that the high N_2_O emission potential of dent corn Andisol is partly attributed to saprophytic, *nosZ* gene-missing pseudomonad denitrifiers.

Nitrous oxide (N_2_O) is an active greenhouse gas that is responsible for more than 7% of global warming (21) and contributes to the depletion of the ozone layer (28). Global anthropogenic sources of N_2_O include agriculture and industry, from which this gas is generated due to biomass burning, indirect emissions from reactive nitrogen leaching, runoff, and atmospheric deposition (12). The widespread use of nitrogenous fertilizers and manure in agricultural soil is a leading cause of N_2_O emission (6, 22, 29, 32, 45). A recent study demonstrated that 60–70% of the annual anthropogenic N_2_O yield was from agriculture (31), of which nearly 70% of N_2_O gas emission was due to the processes of nitrification and denitrification by soil microorganisms in farm soil (15, 21).

Chemical fertilization using ammonia products, such as ammonium sulfate ([NH_4_]_2_SO_4_) or urea (CO[NH_2_]_2_), often accelerates nitrification, leading to N_2_O emission in upland farming soils (3, 47). Conversely, biological denitrification is another microbial process, otherwise known as nitrate respiration, in which NO_3_^−^ or other oxygenated nitrogen compounds serve as electron acceptors for the degradation of organic substances under anaerobic conditions. In the process of denitrification, nitrate reductase, nitrite reductase, nitric oxide reductase, and nitrous oxide reductase (N_2_OR), encoded by the *napA*/*narG*, *nirS*/*nirK*, *norB*, and *nosZ* genes respectively, are relevant to N_2_O emission from agricultural soils. N_2_O in particular is actively released as the final reduced gas by some N_2_OR-malfunctional, and often *nosZ* gene-missing, denitrifiers (26, 35).

The Shizunai Experimental Livestock Farm of Hokkaido University is a relatively active N_2_O-emitting area in Japan (16). We established a reproducible N_2_O emission assay system using gellan gum soft gel medium, and isolated and characterized culturable N_2_O-emitting eubacteria from the farmland soils of reclaimed tropical peatland (9). In the Andisol farmland in Hokkaido, *Paenibacillus* sp. and a tentative *Leptothrix* sp., both of which showed relatively active N_2_O emission, were directly isolated from a suspension of the thawing pasture topsoil. However, their N_2_O-emitting potentials toward a suspension of the thawing soil itself were parts per thousand (38). We speculated that soil microbial communities in farmed corn soil in the summer and autumn are affected not only by plowing and fertilization, but also by the large mass of organic carbons provided by crop roots. Therefore, the saprophytic and facultatively anaerobic bacteria that emerged in sugarless soft gel medium were further examined for active N_2_O emission, which resulted in the isolation of 10 bacteria that showed active N_2_O emission potentials almost equivalent to the soil suspension.

In this study, we present the physiological and genetic characteristics of the N_2_O-emitting bacteria isolated from pre-tilled and post-harvest vitric Andisol of a corn farm in Hokkaido, Japan.

## Materials and Methods

### Soil samples

Soil samples (approximately 10–50 g each) were collected from the Hokkaido University Shizunai Livestock Farm in Hokkaido, Japan (42°26′ N, 142°28′ E) after the corn harvest in November 2011 and the following spring before tilling and sowing in April 2012. With the untreated control as neither fertilizer nor manure (CC), 4 treatments had been applied to the corn field; fertilizer (05CF), manure (beef cattle manure with bedding litter) (05CM), and fertilizer and manure (05CFM), all of which were deposited from 2005 to the present date. The relatively newly converted grassland to corn farm (10CFM) had the following farm management history: grassland between 2004 and 2009 was tilled on November 30, 2009, and was then converted to a corn farm from 2010 to the present date, with fertilizer and manure being deposited from 2010. The fertilizer application rates were 104 kg ha^−1^ N (80 kg ha^−1^ ammonium-nitrogen and 24 kg ha^−1^ urea), 144 kg ha^−1^ P_2_O_5_, and 80 kg ha^−1^ K_2_O. Manure (beef cattle manure with bedding litter) application rates were 215 kg ha^−1^ N, 397 kg ha^−1^ P_2_O_5_, and 325 kg ha^−1^ K_2_O. At the same time, we collected soil samples from cultivated pasture plots that had been treated. Pasture samples were labeled as PC (control), PF (fertilizer), and PFM (fertilizer and manure). The fertilizer application rates were 66.1 kg ha^−1^ N, 175 kg ha^−1^ P_2_O_5_, and 100 kg ha^−1^ K_2_O. Manure application rates were 95.5 kg ha^−1^ N, 74.8 kg ha^−1^ P_2_O_5_, and 164.6 kg ha^−1^ K_2_O. Forty-eight soil samples were obtained from each plot at three points and two different depths, 5 cm (4–6 cm) and 15 cm (14–16 cm), in November 2011 and April 2012, respectively. Samples were obtained before tillage with a rotary cultivator and kept in zippered plastic bags at 4°C until used.

### N_2_O emission assay for soil suspensions under an alternative N source

**Fig. 1:**
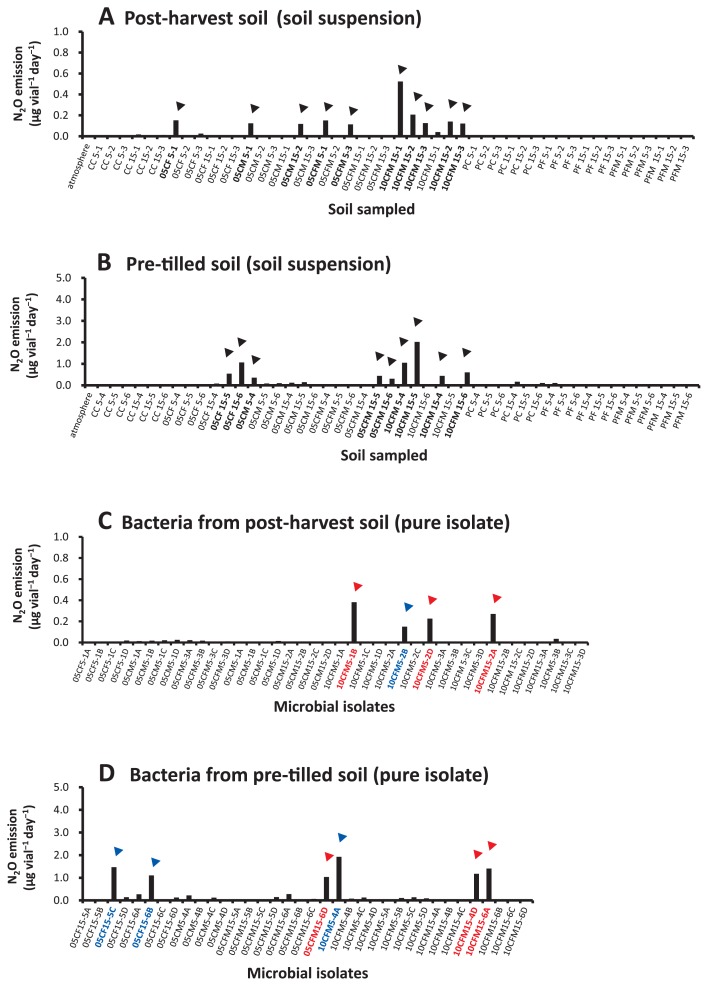
N_2_O emission potential of soil microbial communities from corn farm Andisol, and bacteria isolated from culture media. N_2_O emission from soil suspensions incubated in gellan gum soft-gel media (pH 5.0) with 500 mg L^−1^ NO_3_^−^ or 500 mg L^−1^ NH_4_^+^ at 20°C for 7 d in the dark without sucrose supplementation. Soil samples were collected from farms (autumn 2011) for the preparation of inoculants. KNO_3_ was used as a substrate (A). N_2_O emission by bacteria isolated from culture media in A (B). Soil collected from the farms (spring 2012) were inoculated using KNO_3_ as the substrate (C). N_2_O emission by bacteria isolated from culture media in C (D). Note that the scales of the y-axes in C and D are 5-fold larger than those of the others. Samples marked with the symbol (▼) were further screened for N_2_O emitters. Blue-colored (▼) and red-colored (▼) symbols in C and D are *nosZ*-harboring and *nosZ*-missing denitrifiers respectively.

In order to investigate the main causative microorganisms for N_2_O production in soil in autumn (post-harvest) and spring (pre-tilled), two media for the culture-based N_2_O emission assay were used alternatively. Since mineral N is the substrate for N_2_O production, an excess concentration of (NH_4_)_2_SO_4_ (500 mg L^−1^ N, as 2.4 g L^−1^ of [NH_4_]_2_SO_4_) or KNO_3_ (500 mg L^−1^ N, as 3.6 g L^−1^ of KNO_3_) was added to Winogradsky’s mineral solution separately with 0.01% (w/v) CaCO_3_ (8, 38). The pH of the solution for the enrichment culture was adjusted to 5.0 with 1 M aqueous solution of H_2_SO_4_, followed by filtration through a polytetrafluoroethylene (PTFE) membrane (pore size, 0.45 μm) in order to remove insoluble mineral salts. Gellan gum powder 0.3% (w/v) was added as the gel matrix to the resulting solution. The mixture was heated at 117°C for 15 min, and mixed well as the mixture liquefied (7). After cooling to room temperature, 10.0 mL medium was poured into a 30-mL gas chromatographic vial (Nichiden-Rika Glass Co., Kobe, Japan), sealed with a butyl rubber plug and screw cap septum, and autoclaved at 121°C for 15 min. The headspace volume of the vial containing 10.0 mL medium was 22.6 mL (8). We further compensated the headspace volume as 22.5 mL due to considerations of the inoculant volume (generally 100 μL) unless otherwise stated. The medium used in the preliminary culture-based N_2_O emission assay to screen N_2_O emitters (Figs. 1 and S1) did not contain any additional sugar, while 1.5 or 15 mM sucrose-containing medium was used for other N_2_O assays.

Soil suspensions were prepared by adding 10 mg of fresh soil to 10 mL of sterilized water, vortexing for 1 min, and standing for 10 min. In each sample, a 100-μL aliquot of the supernatant was inoculated into 10.0 mL soft gel medium and vortexed for 1 min. The vials were incubated in the dark at 20°C for 7 d unless otherwise stated. The headspace gas in each vial was sampled with a 1-mL gas tight syringe (Pressure Lock Series, VICI Precision Sampling Inc., Baton Rouge, LA, USA) and analyzed with a gas chromatograph (GC) (Shimadzu GC-14B, Kyoto, Japan). The GC was equipped with an electron capture detector (ECD) (Shimadzu ECD-2014) kept at 340°C using a 1-m Porapak N column (Waters, Milford, MS, USA) to be held in 60°C, with a carrier gas of Ar with 5% CH_4_.

### Isolation of and screening for N_2_O-emitting bacteria

Culture medium from which N_2_O was actively produced during the N_2_O emission assay was used as the source in the isolation of N_2_O-emitting bacteria. A 100-μL aliquot of the suspension (medium diluted 10,000-fold with sterilized water) was inoculated into modified Winogradsky’s gellan gum plates (MWG, Winogradsky’s mineral mixture with 0.5% sucrose as the carbon source, 500 mg L^−1^ N as the nitrogen source (3.6 g KNO_3_ L^−1^), pH 5.0 with H_2_SO_4_, and 2% gellan gum for the gel matrix). Plates were incubated at 20°C in the dark for 4 d. The dominant bacterial colonies apparent on the MWG plates were isolated and purified several times on MWG plates. In order to examine N_2_O emitters, two loops of the isolated bacterial colonies were inoculated on soft gel medium without supplementation with sugar and vortexed for 30 s. All vials were analyzed by ECD-gas chromatography after incubating at 20°C in the dark for 7 d, at which point the concentration of N_2_O in the headspace gas reached its maximum level. When the headspace gas (a volume of 22.5 mL) contained 1 μL L^−1^ of N_2_O (1 ppmv, equivalent to 2.0 μg L^−1^), the absolute amount of N_2_O produced from the culture medium per vial was 45.2 ng. The absolute amount of N_2_O in the headspace was simply divided by the incubation days, leading to N_2_O emitted d^−1^ from the 10-mL culture (as ng d^−1^ or μg d^−1^).

### Extraction of DNA from N_2_O emitters and 16S rRNA gene amplification

The bacterial isolates recognized as active N_2_O-emitting bacteria were cultured overnight on a shaker at 110 rpm in 50 mL LB medium at 20°C in the dark. Cultured medium was transferred to a sterilized 50-mL Falcon tube and centrifuged at 8,000 × *g* for 10 min in order to obtain a pellet of bacterial cells. The cells, washed with sterile water and re-suspended in 1.5 mL TE buffer, were used for DNA extraction using the Isoplant II DNA Extraction Kit (Wako, Osaka, Japan). Using isolated DNA as the template, the 16S rRNA genes were amplified by PCR (EX-*Taq* polymerase, Takara, Otsu, Japan) on a Takara PCR Thermal Cycler Dice TP600 with its universal primer pair *27F/1525R* (5′-AGA GTT TGA TCC TGG CTC AG-3′/5′-AAA GGA GGT GAT CCA GCC-3′).

The PCR conditions used were as follows: preheat at 95°C for 5 min, denature with 35 cycles at 95°C for 30 s, anneal at 55°C for 30 s, extend at 72°C for 30 s, and complete at 72°C for 7 min. The second amplification of sequencing PCR used 3 forward (*27F*, *341F*, *1112F*) and 3 reverse (*803R*, *1080R*, *1492R*) primers (17, 41, 43). In the direct PCR sequence analysis, an ABI Prism 310 genetic analyzer was used with a BigDye Terminator version 3.1 cycle sequencing ready-reaction kit (Applied Biosystems, Foster City, CA). The conditions for sequencing PCR were 25 cycles at 96°C for 30 s for denaturation, at 50°C for 15 s for annealing, and at 60°C for 4 min for extension. The determined sequence was searched in the BLASTN database program provided by the DDBJ (DNA Data Bank of Japan, National Institute of Genetics, Mishima, Japan) or NCBI (National Center of Biotechnology Information, USA).

### Optimal conditions for N_2_O production

In order to evaluate the effects of a supplemented carbon source on N_2_O emitters, a series of sucrose concentrations (0, 1.5, and 15 mM) were added to 10 mL of Winogradsky’s medium supplemented with 5 mg KNO_3_ (N supplement). *Pseudomonas* sp. was cultured on a shaker at 110 rpm in 50 mL of Winogradsky’s medium supplemented with 15 mM sucrose at 20°C for 24 h in the dark in order to obtain fresh inoculates for the N_2_O emission assay. Inoculates were collected from 50 mL culture medium by centrifuging at 8,000 ×*g* at 4°C for 10 min, washed with Milli-Q water several times, and then re-suspended in sterilized water. The suspension of bacteria (10^6^ cells mL^−1^) was added to Winogradsky’s medium (10 mL) in 30-mL gas chromatographic vials. The initial medium containing 1.5 mM sucrose was used for further incubations in order to analyze optimum pH and N_2_O emission. After a 7-d incubation at 20°C in the dark, the headspace gas was analyzed with the GC.

The pH values of media before and after culturing were recorded in order to identify any marked changes in pH during the incubation of the test bacteria. Adjustments in pH (3.5–7.6) were made with 1 M H_2_SO_4_ and 1 M KOH solutions before autoclaving. The optimal pH was measured using the portable pH meter Horiba F-22 (Horiba, Kyoto, Japan) connected to an Orion 8013BN glass electrode (Orion, Beverly, MA, USA). pH was measured in isolates from spring soil before tilling using the hand-held pH meter, the B-212 Twin Compact pH meter (Horiba). Four isolates (10CFM5-1B and 10CFM5-2D from post-harvest soil and 10CFM5-4A and 10CFM15-6A from pre-tilled soil) were used in this experiment.

### Acetylene-blocking assay of N_2_O-emitting bacteria

In order to investigate the effects of acetylene on N_2_O emitters, N_2_O-emitting bacteria were inoculated into 10 mL soft gel medium in the same gas chromatographic culture vials as those used in the N_2_O emission assay, and pure acetylene gas (2.25 mL) was injected into the headspace (22.5 mL), giving a concentration of 10% acetylene (1). In order to allow excess gas to escape during the injection of acetylene, a sterile needle was inserted by penetrating the butyl rubber plug. Cultured vials with inoculates, but without an injection of acetylene gas were prepared as controls. Treated samples and controls were examined in triplicate. After an incubation for 5–7 d, the amount of N_2_O in the headspace gas was measured using ECD-gas chromatography (Shimadzu GC-14B equipped with Shimadzu ECD-2014) as described above.

### Detection of the *nosZ* gene from N_2_O-emittable *Pseudomonas* spp. by PCR

In denitrifying pseudomonads, *nosZ* was detected by PCR, using the primer set *nosZ-1111F* (5′-STA CAA CWC GGA RAA SG-3′), *nosZ-661F* (5′-CGG CTG GGG GCT GAC CAA-3′), *nosZ-1527R* (5′-CTG RCT GTC GAD GAA CAG-3′), and *nosZ-1773R* (5′-ATR TCG ATC ARC TGB TCG TT-3′) (33). The PCR conditions used were as follows: preheating at 95°C for 5 min, 35 cycles of denaturation at 95°C for 30 s, annealing at 50°C for 30 s, and extension at 72°C for 30 s. The reaction was completed at 72°C for 7 min. Sequencing of the PCR amplicons assignable as *nosZ* fragments was detected by agarose gel electrophoresis. If one bacterium gave an amplicon, its sequence determination followed by a homology search on the DNA database (NCBI) was performed in order to confirm whether the bacterium possessed the *nosZ* gene in its genome.

### Detection of *narG* and *nirS* genes from pseudomonad denitrifiers

A PCR assay was also performed for the *narG* and *nirS* genes, which encode the α-subunit of membrane-bound, respiratory nitrate reductase and nitrite reductase containing cytochrome-cd_1_, respectively, in order to determine whether denitrification is linked to these reductoxidases. Regarding the specific detection of the *narG* gene, the conserved domain of the target enzyme was surveyed for the universal primer design. Amino acid sequences converted from the base sequence of the *narG* gene encoding the nitrate reductase α-subunit (NarG) were randomly collected from *Alphaproteobacteria*, *Betaproteobacteria*, *Gammaproteobacteria*, *Firmicutes*, *Actinobacteria*, and some other minor phyla. Among the 50 eubacterial sequence data for NarG, two conserved regions were selected in more than HYVGQEK (positions of amino acids at 567–573 for the nitrate reductase α-subunit of *Pseudomonas fluorescens* AK15 with accession no. U71398.1 (25) and DMHPFIH (positions at 792–798 for the same protein) for the design of the degenerated forward (5′-CAY TAY GTS GGS CAR G-3′) and reverse (5′-TGD ATR AAN GGR TGC A-3′) primers, respectively. A 606-bp amplicon (LC034247.2) was obtained from *Pseudomonas* sp. 05CF15-5C, and using this sequence, the secondary primers *narG-2152F* (5′-TCG GGC AAG GGC CAT GAG TAC-3′) and *narG-2332R* (5′-TTT CGT ACC AGG TGG CGG TCG-3′) for the shorter amplicon (approx. 200 bp) were further designed. Conversely, the partial *nirS* gene was amplified using a pair of known primers, *nirSCd3Af* (5′-AAC GYS AAG GAR ACS GG-3′) and *nirSR3cd* (5′-GAS TTC GGR TGS GTC T-3′) (40).

### Phylogenetic analysis of N_2_O-emitting bacteria for the 16S rRNA gene

In the phylogenetic analysis of these 10 N_2_O-emitting *Pseudomonas* isolates, the 1456-bp sequences obtained for their 16S rRNA genes in the region from positions 74 to 1529 for *Escherichia coli* (accession no. J01859.1) were analyzed by the maximum composite likelihood estimation, along with 9 reference species selected from several groups of pseudomonads registered in the NCBI DNA database. This neighbor-joining tree method was conducted for the identification of pseudomonads at the species level using the computing tool for a phylogenetic analysis in Molecular Evolutionary Genetics Analysis (MEGA v. 6.06) software (39). Under an estimation of distances between all pairs of sequences with 1,000 bootstrap replicates, clustering of the complete denitrifiers and incomplete denitrifiers was performed and used for further determinations of their emergence in corn farm Andisol.

### Taxonomic classification of the soil bacterial community using a 16S rRNA metagenomic analysis

A metagenomic analysis for the soil bacterial community structure was performed using the next-generation sequencing system Ion Torrent PGM (Thermo Fisher Scientific, Waltham, MA USA) targeting the 16S rRNA gene. 16S rRNA metagenomic libraries were prepared following the instructions provided by the Ion 16S metagenomics kit (Life Technologies, CA, USA). Bacterial 16S rRNA gene regions were amplified by the Ion 16S metagenomics kit using the primer sets V2-4-8 and V3-6,7-9 on a 30-μL scale. PCR amplification was performed under the following conditions: an initial activation step was conducted at 95°C for 10 min, denaturing at 95°C for 30 s, annealing at 58°C for 30 s, and extension at 72°C for 20 s was repeated for 30 cycles, and the final extension was performed at 72°C for 7 min. The PCR sample amplified using V2-4-8 was mixed with an equal volume of the other sample amplified with V3-6,7-9, and the mixture thereof was subjected to purification with Agilent AMPure XP (Agilent Technologies, Santa Clara, CA, USA) according to the instructions provided. The PCR products thus purified were quantified by the Qubit dsDNA HS Assay Kit (Thermo Fisher Scientific).

The DNA libraries of the 16S rRNA gene were prepared using the Ion Plus Fragment Library Kit (Life Technologies) and barcoded with the IonXpress Barcode Adapters 1–16 Kit (Life Technologies) in order to distinguish 7 samples of soil DNA with barcode numbers 1 to 7. Adapter-ligated and nick-repaired DNA chains were subjected to library amplification under the following conditions: 95°C for 5 min, 6 cycles at 95°C for 15 s, at 58°C for 15 s, and at 70°C for 1 min. The bioanalyzer with the Agilent High Sensitivity DNA kit (Agilent Technologies) was used to assess the quality of the libraries. Each DNA library from soil samples was diluted to 26 pM, subjected to emulsion PCR in order to amplify the sequencing template onto Ion Sphere Particles (ISPs) with the Ion OneTouch 2 system (Life Technologies), and enriched with the Ion OneTouch ES using the Ion PGM Template OT2 400 Kit (Life Technologies) according to the manufacturer’s protocol. Enriched ISPs were subjected to single-end sequencing on Ion Torrent PGM (Life Technologies) using the Ion PGM Sequencing 400 kit (Life Technologies) with Ion 316 Chip v2 (Life Technologies) for 850 flows.

All sequences were processed for data analysis using Ion Torrent platform-specific pipeline software, Ion Reporter 5.0 16S Metagenomics Workflow (Life Technologies) automatically accessible to MicroSEQ 16S Reference Library v2013.1 (Thermo Fisher Science), and Greengenes v13.5 (The Greengenes Database Consortium, http://greengenes.secondgenome.com/). The number of copies needed for matching was set at 10.

### Nucleotide sequence accession numbers

The sequences of the 16S rRNA genes and denitrification-related genes from N_2_O-emitting *Pseudomonas* isolates have been deposited in the GenBank/DDBJ databases. The accession numbers of the 16S rRNA genes from *Pseudomonas* isolates are AB856847–AB856850 (from post-harvest soil) and LC007966–LC007971 (from pre-tilled soil). LC047837–LC047840 are those of the partial *nosZ* gene, while LC047828–LC047836 and LC034243–LC034252 are those of the partial *nirS* and *narG* genes, respectively.

## Results

### N_2_O-emitting capacities of soil microbial community members in post-harvest and pre-tilled corn farm Andisol

The N_2_O-emitting capacities of culturable soil bacteria from post-harvest and pre-tilled corn farm Andisol were examined; the supernatant of each soil suspension was inoculated on 0.3% gellan gum soft gel medium in closed glass vials. Forty-eight and 44 soil samples obtained from post-harvest and pre-tilled farmland, respectively, were incubated in medium supplemented with an alternative mineral nitrogen source (NO_3_^−^ N or NH_4_^+^ N) in the absence of sucrose (Figs. 1 and S1). Some of the inoculants displayed particularly active N_2_O emission, greater than 0.1 μg vial^−1^ d^−1^, in the headspace gas (equivalent to 15 ppmv N_2_O in the headspace gas after 7 d) with the addition of NO_3_^−^ N. The most active Andisol suspension culture produced 2.0 μg vial^−1^ d^−1^ of N_2_O (soil sample 10CFM15-5). All active cultures were from fertilized corn farm soil (10CFM, 05CFM, or 05CF) and not pasture soils (Fig. 1A–D).

### N_2_O emitters isolated from farm soils

Among the ten soft gel cultures of the post-harvest soil microbiota and nine cultures of the pre-tilled soil microbiota that showed relatively high active N_2_O emission, the major emergent bacterial colonies of each culture, appearing as the 1^st^ to 4^th^ highest population size on a plate, were isolated and purified several times on MWG plates (Fig. 1A and B). We subsequently screened 76 microbial isolates from N_2_O-producing cultures, and the N_2_O emission potential of each bacterial isolate was determined. Among the 40 microbial community members obtained from the culture of post-harvest soil suspensions, four isolates exhibited N_2_O-producing activity greater than 0.15 μg N_2_O vial^−1^ d^−1^, and the most active N_2_O emitter, isolate 10CMF5-1B, produced 0.38 μg N_2_O vial^−1^ d^−1^ (Fig. 1C). Among the 36 microbes obtained from pre-tilled soil cultures, six isolates exhibited higher N_2_O emission than those selected from post-harvest soil bacteria (more than 1.0 μg N_2_O vial^−1^ d^−1^). The most active isolate 05CFM15-4A showed a performance of 1.9 μg N_2_O vial^−1^ d^−1^ (Fig. 1D). All active N_2_O-emitting bacteria screened from post-harvest and pre-tilled soil were identified as Gram-negative bacteria of the genus *Pseudomonas* (Table 1), which includes some well-known denitrifiers active as N_2_O emitters (4, 27).

### Identification of N_2_O-emitting pseudomonads by a phylogenetic analysis of 16S rRNA gene sequences

**Fig. 2:**
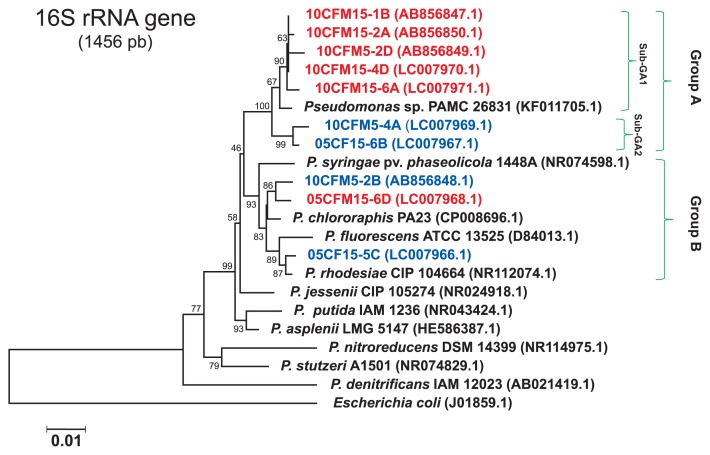
Phylogenetic position of N_2_O-emitting *Pseudomonas* species isolated from a corn farm Andisol suspension culture. The phylogenetic relationships among the N_2_O-emitting *Pseudomonas* sp. obtained from chemically fertilized corn farmland soil were computed, along with 10 reference species selected from several groups of pseudomonads. This neighbor-joining tree was deduced based on the 16S rRNA gene sequences obtained of the 1456-bp region using the maximum composite likelihood method for the estimation of distances between all pairs of sequences simultaneously with 1,000 bootstrap replicates by the Kimura 2-parameter model. The scale bar represents 0.01 substitutions per nucleotide site. The same region of the sequence from *E. coli* (J01859.1) was used as the outgroup. *nosZ*-missing denitrifiers are shown by red characters and *nosZ*-harboring denitrifiers by blue characters.

The 16S rRNA gene sequences of the *Pseudomonas* denitrifiers, 10CMF5-1B, 10CMF15-2D, and 10CMF5-2A from post-harvest soil and 10CFM15-4D and 10CFM15-6A from pre-tilled soil formed a clade (group A). Group A was separated into two sub-groups, sub-group A1 for the five *nosZ*-missing pseudomonads and sub-group A2 for the two *nosZ*-harboring *Pseudomonas* denitrifiers 10CMF5-4A and 05CF15-6B (Fig. 2). *Pseudomonas* sp. PAMC 26831 (accession no. KF011705), which had been isolated from subarctic Alaskan grassland soil by Park and Kim (24), showed the highest sequence homology to members in sub-group A1. The other *nosZ*-harboring denitrifiers *Pseudomonas* sp. 10CMF5-2B and 05CF15-5C made another clade (group B) distinguishable from pseudomonads in group A. Group B consisted of the *nosZ*-missing denitrifier *Pseudomonas* sp. 05CFM15-6D from pre-tilled soil and some reference pseudomonads, including *P. fluorescens* (Fig. 2).

### Response of N_2_O-emitting pseudomonads to supplemented sucrose in the production of N_2_O

**Fig. 3:**
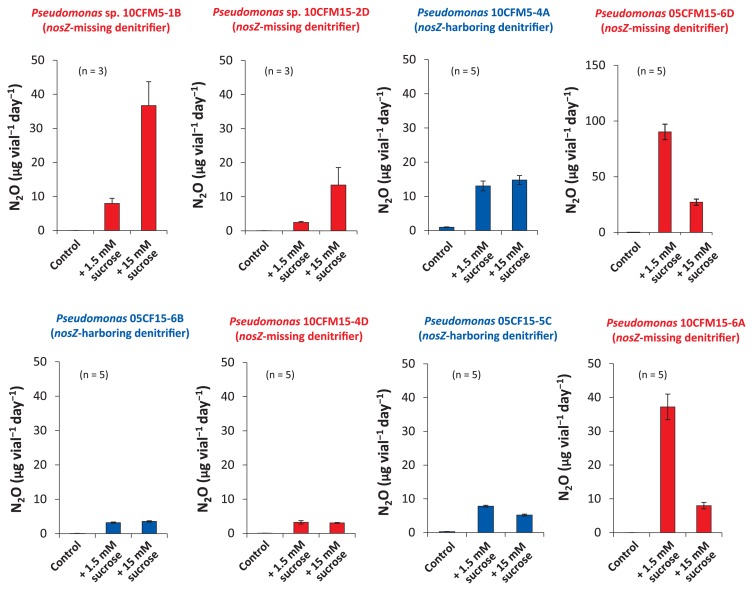
Effects of sucrose supplementation on N_2_O emission by N_2_O-emitting *Pseudomonas* isolates. Winogradsky’s gellan gum medium contained 3.6 mg mL^−1^ KNO_3_ and was supplemented with sucrose (1.5 mM and 15 mM). An incubation was performed at 20°C for 4 d in the dark. Values are represented as means ± standard deviation (SD), as shown by error bars. The red columns are those of N_2_OR-missing N_2_O emitters (tentatively defined as *nosZ*-missing denitrifiers), and the blue columns are those of the *nosZ*-harboring N_2_O emitters. Note that the scale of the y-axis in *Pseudomonas* sp. 05CFM 15-6D is 3-fold larger than those of the others. Values are represented as means ± standard deviations (SD), as indicated by error bars (n=3). Asterisk * indicates *p*<0.01 by the Student’s *t*-test.

Since most denitrifiers are saprophytes and *Pseudomonas* bacteria are generally heterotrophic, the responses of N_2_O-emitting pseudomonads to carbon source-rich conditions were investigated. The addition of sucrose as a carbon source to standard assay medium led to an increase in the production of N_2_O by the N_2_O emitters, *Pseudomonas* 10CFM5-1B and 10CFM5-2D (Fig. 3). N_2_O production from culture medium without the addition of sugar was not significant (less than 2.0 μg N_2_O vial^−1^ d^−1^) when test bacteria were cultured in sugarless medium, whereas N_2_O production was approximately 20-fold higher in the presence of 1.5 mM sucrose, which produced 8.0 μg N_2_O vial^−1^ d^−1^. N_2_O emission from *Pseudomonas* spp. 10CFM5-1B cultured in 15 mM sucrose-supplemented medium was more than 4-fold higher than that incubated in 1.5 mM sucrose-supplemented medium, reaching 37 μg N_2_O vial^−1^ d^−1^.

In contrast, two N_2_O-emitting pseudomonads from pre-tilled soil (isolates 10CFM15-6A and 05CFM15-6D) also showed strong responses to 1.5 mM sucrose-supplemented medium; however, N_2_O emission markedly decreased upon supplementation with a 10-fold higher concentration of sucrose (Fig. 3). The responses of these N_2_O emitters to additional sucrose suggested that the carbon source is an important factor for active N_2_O emission due to the active cell growth of the highly heterotrophic and denitrifying *Pseudomonas* spp. and active nitrate respiration. Among these N_2_O emitters, *Pseudomonas* sp. 10CFM15-6A cultured in 1.5 mM sucrose-supplemented medium showed the most active production of N_2_O (37 μg N_2_O vial^−1^ d^−1^).

### Acetylene-blocking assay and PCR assay for the detection of N_2_OR activity and/or the *nosZ* gene

**Fig. 4:**
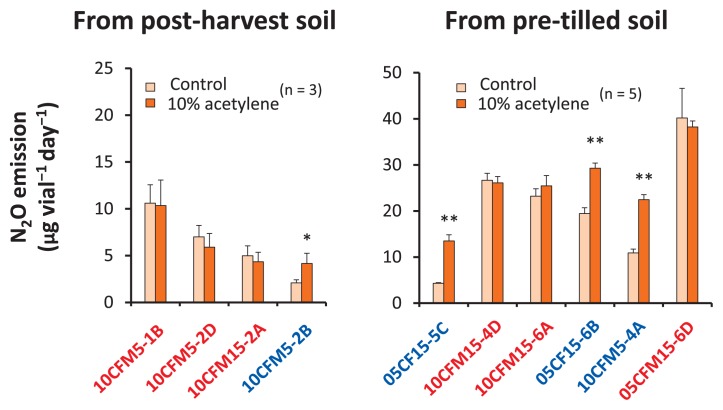
Acetylene-blocking assay for N_2_O-emitting *Pseudomonas* isolates N_2_O production by N_2_O-emitting *Pseudomonas* sp. in Winogradsky’s gellan gum medium supplemented with excess KNO_3_ (3.6 mg mL^−1^ KNO_3_) and 1.5 mM sucrose was examined in the presence or absence of 10% acetylene. Following an incubation at 20°C for 4 d in the dark, the absolute amount of N_2_O was quantified. The levels of N_2_O are represented as means ± SD. Single and double asterisks (* and **) indicate significant differences (*p*<0.05 and 0.01 respectively) by the Student’s *t*-test. Note that the scale of the y-axes in bacteria from pre-tilled soil is 2-fold larger than that of post-harvest soil.

The addition of 10% acetylene did not result in any significant acceleration in the production of N_2_O by the *Pseudomonas* isolates 10CFM5-1B, 10CFM5-2D, 10CFM15-4D, 10CFM15-6A, and 05CFM15-6D, suggesting that these isolates are incomplete denitrifiers that are almost missing N_2_O-reducing activity to produce N_2_ gas (Fig. 4). Conversely, *Pseudomonas* isolates 10CFM5-2B, 05CF15-5C, 05CF15-6B, and 05CFM5-4A demonstrated significantly higher (approximately 2-fold) N_2_O emission in the presence of acetylene in the headspace (*p*<0.001), and, thus, these isolates were characterizable as complete denitrifiers that possess a functional *nosZ* gene.

In the PCR assay to detect the *nosZ* gene toward N_2_O-emitting pseudomonads, four isolates, 10CFM5-2B, 05CF15-5C, 05CF15-6B, and 10CFM5-4A, gave amplicons of 850–900 bp (Fig. S2). All the PCR products sequenced were confirmed to be *nosZ* fragments by a homology search on the DNA database (NCBI). All positive pseudomonads in the *nosZ-*PCR assay also showed weak, but positive responses in the acetylene-blocking assay, proving that they are complete denitrifiers. Unlike the *nosZ* gene, the *narG* gene was detected from all N_2_O-emittable *Pseudomonas* spp. reported in this study (Fig. S3).

### Optimum pH for N_2_O emission by *Pseudomonas* denitrifiers

The optimum pH of the *Pseudomonas* denitrifiers 10CFM5-1B (*nosZ*-missing denitrifier) and 10CFM5-2B (*nosZ*-harboring denitrifier) from post-harvest soil for N_2_O production was measured in a series of pre-autoclaved media with a range of pH values (3.6–7.6). The pH values in medium slightly increased after autoclaving, but decreased after the incubation (38), while the pH values in medium remained nearly in the original region, even though it contained 1.5 mM sucrose (Fig. S4). Both of the N_2_O emitters, *nosZ*-missing *Pseudomonas* 10CFM5-1B and *nosZ*-harboring *Pseudomonas* 10CFM5-2B, produced N_2_O at an appropriate pH range after culturing (5.8–6.3 and 6.0–6.8, respectively). *Pseudomonas* sp. 10CFM5-1B showed a strong response at approximately pH 6.0 during the incubation, while *nosZ*-harboring 10CFM5-2B actively emitted N_2_O in a range of 6.2–6.8.

**Fig. 5:**
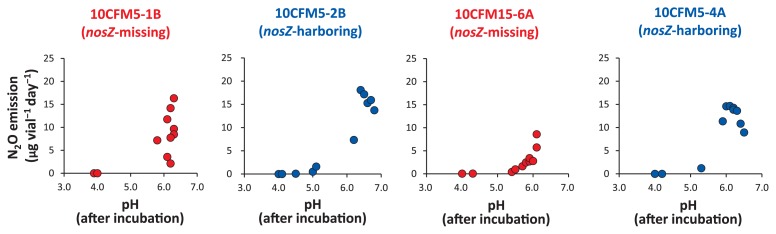
Response of four N_2_O-emitting bacteria to varying pH in medium. The responses of four N_2_O-emitting *Pseudomonas* bacteria, isolates 10CFM5-1B, 10CFM5-2B, 10CFM15-6A, and 10CFM5-4A, to various pH (3.8–7.7) values before incubations (y-axes on the bottom panels A–D) were tested in gellan gum soft gel medium supplemented with 5 mM KNO_3_ and 1.5 mM sucrose. Note that the pH values in the x-axes are those maintained after a 7-d incubation (pH, after incubation). Red-colored characters for the isolate codes and the marks are *nosZ*-missing isolates, while the blue ones are those of *nosZ*-harboring isolates.

We also investigated the optimum pH of two more *Pseudomonas* denitrifiers 10CFM5-4A (*nosZ*-harboring) and 10CFM15-6A (*nosZ*-missing) from pre-tilled soil for N_2_O production at a range of pH values after the incubation (Fig. 5). The responses of *nosZ*-harboring 10CFM5-4A and *nosZ*-missing 10CFM15-6A to medium pH were both similar to the isolates from post-harvest soil.

*nosZ*-missing 10CFM15-1B and 10CFM15-6A both exhibited the ability to converge the medium pH at 6.0–6.1, in which medium increments in the final pH from 5.9 to 6.1 showed proportional increases in the production of N_2_O and optimum pH for the active production of N_2_O appeared to be at pH 6.1 or higher. Emission decreased gradually in the acidic region and was nearly zero at pH 5.1 or lower. Cell growth was very active in all media showing high turbidity. Similarly, an inverse relationship was observed between N_2_O production and acidity in the pH range of 5.1–6.4 and 5.3–6.1, respectively, in two *nosZ*-harboring pseudomonads 10CFM5-2B and 10CFM5-4A. This was dissimilar to N_2_O emitters that have adapted to medium-strongly acidic tropical peat soils (*e.g.*, *Janthinobacterium* sp.) (9), but more similar to that of an active N_2_O emitter isolated from the Andisol of an unfertilized pasture (tentative *Leptothrix* sp. of subclass *Betaproteobacteria*) (38).

### Detection of denitrification genes (*narG*, *nirS*, and *nosZ*) and each phylogenetic relationship among nosZ-harboring *Pseudomonas*

In the PCR assay for the detection of the *nosZ* gene among N_2_O-emitting pseudomonads, four isolates, 10CFM5-2B, 05CF15-5C, 05CF15-6B, and 10CFM5-4A, produced amplicons of 700–900 bp (Fig. S3). All pseudomonads with positive *nosZ*-PCR also showed positive responses to the acetylene-blocking assay, proving that they are denitrifiers with relatively low N_2_OR activity (Table 1).

Unlike the *nosZ* gene, the *narG* gene was detected in all pseudomonad denitrifiers using the PCR assay (Fig. S3). A phylogenetic analysis using the 204-bp sequence of their *narG* genes in the region from positions 2152 and 2355 for *P. fluorescens* AK15 (accession no. U71398.1) along with some reference species of pseudomonads showed phylogenetic relationships among the 10 N_2_O-emitting *Pseudomonas* bacterial isolates. A dendrogram of the partial *narG* gene was similar to that obtained by the 16S rRNA gene analysis (Fig. 2). Furthermore, all isolates also harbored the *nirS* gene, except for *Pseudomonas* 10CFM5-2B, which possessed the *nirK* gene (data not shown). A phylogenetic analysis of the 366-bp sequence of the *nirS* gene in the region of positions 937 to 1316 for *P. stutzeri* (accession no. AAZ43111.1) showed a dendrogram that was very agreeable with that of the 16S rRNA gene-based phylogenetic tree (Fig. S5).

**Fig. 6:**
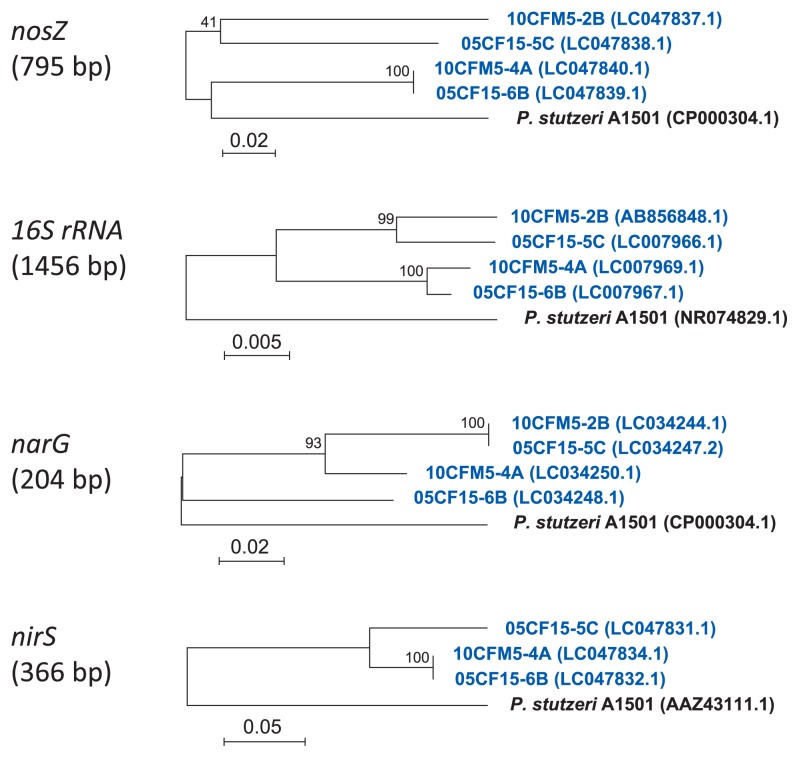
Phylogenetic analysis of *narG*, *nirS*, and *nosZ* genes of *Pseudomonas* denitrifiers. The phylogenetic relationships among *nosZ*-harboring N_2_O-emitting *Pseudomonas* isolates were computed, along with *P. stutzeri* A1501 as a reference species of a pseudomonad. Regarding the partial *nosZ* gene sequence of the 795-bp fragment, a neighbor-joining tree was deduced under the same analytical conditions as those for the 16S rRNA gene-based phylogeny. The scale bar (0.02 and 0.005 for *nosZ* and *16S rRNA*, respectively) represents substitutions per nucleotide site. The partial sequences of the *narG* (204-bp) and *nirS* (366-bp) genes were also subjected to the construction of a neighbor-joining tree under the same conditions. *P. stutzeri* A1501 was used as a reference bacterium similar to the same region of each gene-based phylogeny. Strains shown by blue-colored characters indicate *nosZ*-harboring denitrifiers from cultures inoculated with Andisol.

A phylogenetic analysis of these *nosZ*-harboring isolates and *P. stutzeri* A1501 as a reference bacterium showed that the partial sequences of the *nirS* and *narG* genes showed similar phylogenetic dendrograms to those drawn on the 1.5-kbp sequences of their 16S rRNA genes. In contrast, the partial sequences of the *nosZ* gene showed a clearly different phylogenetic pattern, in which two isolates 10CFM5-4A and 05CF15-6B of sub-group A2 were markedly closer to *P. stutzeri* than other isolates in group B (10CFM5-2B and 05CF15-5C) (Fig. 6).

### The soil bacterial community structure shown by the 16S metagenomic analysis

Although soil sample storage at 4°C in a zippered plastic bag continued for more than 4 years, stored soil showed some characteristic bacterial compositions according to land management. Fertilized corn farm soil (10CF 15-5) that had shown active N_2_O emission in culture medium had a more characteristic bacterial composition in the order level than unfertilized corn farm soil (CC 15-4) (Fig. S6); however, the order *Pseudomonadales* was minor and no *Pseudomonas* was detected. *Rhodocyclales* (class *Betaproteobacteria*), *Chromatiales* (class *Gammaproteobacteria*), *Nitrospirales* (phylum *Nitrospirae*), *Myxococcales*, *Desulfuromonadales*, and *Desulfovibrionales* (class *Deltaproteobacteria*), and *Coribacteriales* and *Acidimicrobiales* (phylum *Actinobacteria*) were increased in fertilized corn farm soil. Bacterial community structures in pasture soils (unfertilized PC and fertilized PFM) were clearly distinguishable from corn farm soils. PFM soil contained characteristic bacteria of the orders *Rhizobiales* (class *Alphaproteobacteria*), *Burkholderiales* (class *Betaproteobacteria*), *Chromatiales* (class *Gammaproteobacteria*), and *Gaiellales* (phylum *Actinobacteria*), while the order *Methylophilales* (class *Betaproteobacteria*) was missing in pasture soil.

## Discussion

Regarding the effects of low pH, many biogeological studies have reported that the N_2_O/N_2_ production ratio in denitrification is higher in acidic soil than in neutral and alkaline soil (18, 20, 36, 37), and ample evidence has been obtained in European farm soils (5). Active N_2_O emission in acidic soil is due to incomplete denitrification led by the transient inhibition of N_2_OR for the catalytic reduction of N_2_O into N_2_ (2, 10, 19, 30, 35). Consistent with these findings, our results suggest that one of the most important factors affecting N_2_O production is the weakened or completely lost functionality of N_2_OR necessary for complete denitrification in the microbial community. In nitrate respiration by N_2_OR activity-missing denitrifiers, N_2_O becomes the final electron acceptor instead of N_2_, and a large amount of N_2_O may then be released from soil (13). In the present study, N_2_O-emitting bacteria screened from culturable communities of Andisol showed such traits, and, hence, N_2_OR-inactive traits appear to be more important than the acidic pH of soil for active N_2_O emission from Andisol (Fig. 1).

In the phylogenetic analysis of genus *Pseudomonas* bacteria using the 1456-bp sequence of the 16S rRNA gene in the region of positions 74-1541 for *E. coli* (accession no. J01859.1), the five *Pseudomonas* grouped together in the same clade (Fig. 2) suggest that N_2_OR activity-missing pseudomonads widely inhabit Andisol corn farmland. The members of sub-group A1, five *nosZ*-missing denitrifiers 10CFM5-1B, 10CFM15-2A, 10CFM5-2D, 10CFM15-4D, and 10CFM 15-6A, had a far phylogenetic relationship with common N_2_O-emitting *Pseudomonas* species often containing N_2_OR, such as *Pseudomonas stutzeri* (34) and *Pseudomonas denitrificans* (4, 33). Shina *et al.* (35) reported that *nosZ*-negative or positive is highly affected by soil types; however, our results suggest that the *nosZ*-missing or *nosZ*-harboring trait is strongly associated with genotypes.

A previous study demonstrated that repeated fertilization through the cropping seasons triggered active N_2_O emission from the volcanic Andisol of grassland at Nasu, Tochigi Prefecture, in central Japan. In this non-frozen region, only manure-input grassland actively emitted N_2_O in winter (23). In contrast, in the Shizunai Livestock Farm, Hokkaido Prefecture, the most active annual N_2_O efflux was observed in thawing, pre-tilled corn farm soil in early spring (16, 46). At the transition period (just at thawing) in early spring, NH_4_^+^ concentrations in corn farm soil temporarily increased by approximately 10-fold that of frozen period, and this was followed by the rapid depletion of NH_4_^+^ and accumulation of NO_3_^−^ soon after the thawing event (16). This marked accumulation of NO_3_^−^ in thawing soils and the results of our culturing assay collectively suggest the importance of nitrification-coupled denitrification in active N_2_O emission. Conversely, nitrifier denitrification appears to contribute less to N_2_O emission because microaerobic conditions with the low availability of organic carbon in soils are required for this event (44).

The N_2_O-emitting abilities of the *nosZ*-missing denitrifiers *Pseudomonas* spp. 10CFM5-1B, 10CFM5-2D, 10CMF15-2A, 10CFM15-4D, 10CFM15-6A, and 05CFM15-6D were higher than those of the *nosZ*-harboring *Pseudomonas* spp. 10CFM5-2B, 05CF15-5C, 05CF15-6B, and 05CFM5-4A, particularly in the presence of an additional carbon source (Fig. 3). All the *nosZ*-missing denitrifiers of pseudomonads were present in post-harvest and pre-tilled fertilized soil that had been cultivating corn from 2010 (10CFM), while *nosZ*-harboring denitrifiers were particularly dominant in the pre-tilled spring soil of the corn farm that had been chemically fertilized since 2005 (05CF). The pseudomonads in group A showed the highest sequence homology to *Pseudomonas* sp. PAMC 26831 (accession no. KF011705), which had been isolated from subarctic Alaskan grassland soil by Park and Kim (24), and uncultured *Pseudomonas* sp. RF3-C12 (accession no. JN379403), reported as a catechin degrader in the rhizosphere of *Rhododendron formosanum* (42) (Fig. 2).

*Pseudomonas* N_2_O emitters isolated from post-harvest and pre-tilled corn farm soil appeared to be root-associated because of their obvious saprophytic behaviors, which are highly responsive to supplemented sucrose (Fig. 3). Corn roots in post-harvest soil may enhance carbon and nitrogen turnover by soil microbes and soil ecosystems as reported previously by Henry *et al.* (11), and, accordingly, N_2_O-emitting pseudomonads in pre-tilled farm soil may have survived winter on corn root residues as saprophytes. Furthermore, the neutrophilic properties of these pseudomonads for active N_2_O emission at neutral regions may have been reasonable for rhizoplane bacteria because root surfaces generally maintain neutral pH even in acidic soils (11). In the crop-growing seasons, root-associating, saprophytic pseudomonads may contribute to denitrification in fertilized Andisol corn fields. If denitrifiers were screened under the acetylene-blocking condition, we may have obtained highly N_2_OR-active *Pseudomonas* denitrifiers as key bacteria in order to elucidate the mechanisms underlying passive activity-loss for N_2_OR. Unfortunately, such pseudomonads are mostly diminished from the soil community during long-term (more than 4 years) storage at 4°C (Fig. S6).

The phylogenetic tree of N_2_O-emitting *Pseudomonas* bacteria constructed using sequence variations in their partial *narG* and *nirS* genes sequences is consistent with the dendrogram constructed from the 16S rRNA gene sequences among the pseudomonas. However, the phylogenetic tree of the *nosZ* and 16S rRNA genes among *nosZ*-harboring *Pseudomonas* showed very weak similarities (Fig. 6). Thus, the *nosZ* gene may be particularly unstable among denitrification-associated genes, and easily undergoes the re-construction and deletion of gene regions, leading to the appearance of active N_2_O emitters that have lost N_2_OR activity. It has been hypothesized that *nosZ* is actively transferred by horizontal gene transmission because *nosZ* was previously found to be absent in some bacteria and archaea harboring *nar* and *nir* genes (48). An inspection of a wide range of 16S rRNA trees in the reliable *nosZ* data set was initially performed by Jones *et al.* (14), and they reported that *nosZ* genes in the denitrifiers of *Proteobacteria* were not of a monophyletic origin.

The results of the present study suggest that an important step in the emergence of N_2_O-emitting bacteria in corn farm Andisol is decreased N_2_OR activity; however, these denitrifying *Pseudomonas* bacteria do not solely active N_2_O emission in farmland soil. However, this speculation requires further evidence for the malfunctioning of N_2_OR or absent *nosZ* gene that progressively occurs in proteobacterial denitrifiers in corn farm Andisol, along with a metagenomic analysis on fresh soil from thawing and pre-tilled corn-cultivated farm soil in early spring. Not only the chemical and physical properties of vitric Andisol in the farm, but also its land management history, including cultivated crops as well as manure and chemical fertilizer input, may be key factors responsible for the appearance of such N_2_O-emitting, heterotrophic denitrifiers that have lost N_2_OR activity. These N_2_O emitters of *Pseudomonas* may be a useful bacterial tool to search for effective N_2_O regulation, including delivery methods for functional *nosZ* genes. Hence, further studies on the relationship between N_2_O emitters and vitric Andisol and corn roots in addition to their bias effects during trapping cultures in sugarless gellan gum medium are warranted.

## Supplementary Material



## Figures and Tables

**Table 1:** N_2_O-emitting bacteria isolated from the post-harvest and pre-tilled Andisol of a corn farm and their identification by means of 16S rRNA gene sequence homology.

	Isolated strain	Temporal identification	PCR for *nosZ*^a^	Acetylene-blocking assay^b^	16S rRNA gene (bp)	Accession no.	Type of denitrifier
Post-harvest soil (sampled in 2011)	10CFM5-1B	*Pseudomonas* sp.	−	−	1518	AB856847	*nosZ*-missing
	10CFM5-2D	*Pseudomonas* sp.	−	−	1518	AB856848	*nosZ*-missing
	10CFM15-2A	*Pseudomonas* sp.	−	−	1517	AB856849	*nosZ*-missing
	10CFM5-2B	*Pseudomonas* sp.	+	+	1546	AB856850	*nosZ*-harboring

Pre-tilled soil (sampled in 2012)	05CF15-5C	*Pseudomonas* sp.	+	+	1518	LC007966	*nosZ*-harboring
	05CF15-6B	*Pseudomonas* sp.	+	+	1561	LC007967	*nosZ*-harboring
	05CFM15-6D	*Pseudomonas* sp.	−	−	1518	LC007968	*nosZ*-missing
	10CFM5-4A	*Pseudomonas* sp.	+	+	1577	LC007969	*nosZ*-harboring
	10CFM15-4D	*Pseudomonas* sp.	−	−	1572	LC007970	*nosZ*-missing
	10CFM15-6A	*Pseudomonas* sp.	−	−	1576	LC006671	*nosZ*-missing

a Amplification with a combination of two forward primers (*nosZ-661F* and *1111F*) and two reverse primers (*nosZ-1527R* and *1773R*).

b Acetylene-blocking assay: incubation at 20°C for 7 d with the addition of 1.5 mM sucrose.

The source and identification of ten *Pseudomonas* N_2_O emitters are summarized. In the PCR assay for the detection of the *nosZ* gene, the sequenced amplicon obtained was marked by (+) when it was the targeted gene region. In the acetylene-blocking assay, (−) indicated no significant difference between the treatment and control.
