# Supplementary material for: Physiological and Genotypic Characteristics of Nitrous Oxide (N2O)-Emitting Pseudomonas Species Isolated from Dent Corn Andisol Farmland in Hokkaido, Japan
Source: Microbes Environ. 2016 Apr 22;31(2):93–103. doi: 10.1264/jsme2.ME15155 (PMC4912161; doi:10.1264/jsme2.ME15155)
Supplement: Supplementary file 1 [file 31_93_s1.pdf]

Table S1. Numbers of valid reads obtained by next-generation sequencer

Parameter / Soil	10CMF 15-5	CC 15-4	PMF 15-4	PC 15-6
Number of valid reads	30027	34335	40216	31667
Mapped reads in sample	1992	4033	4217	1923
Un-Mapped reads in sample	13	230	38	0

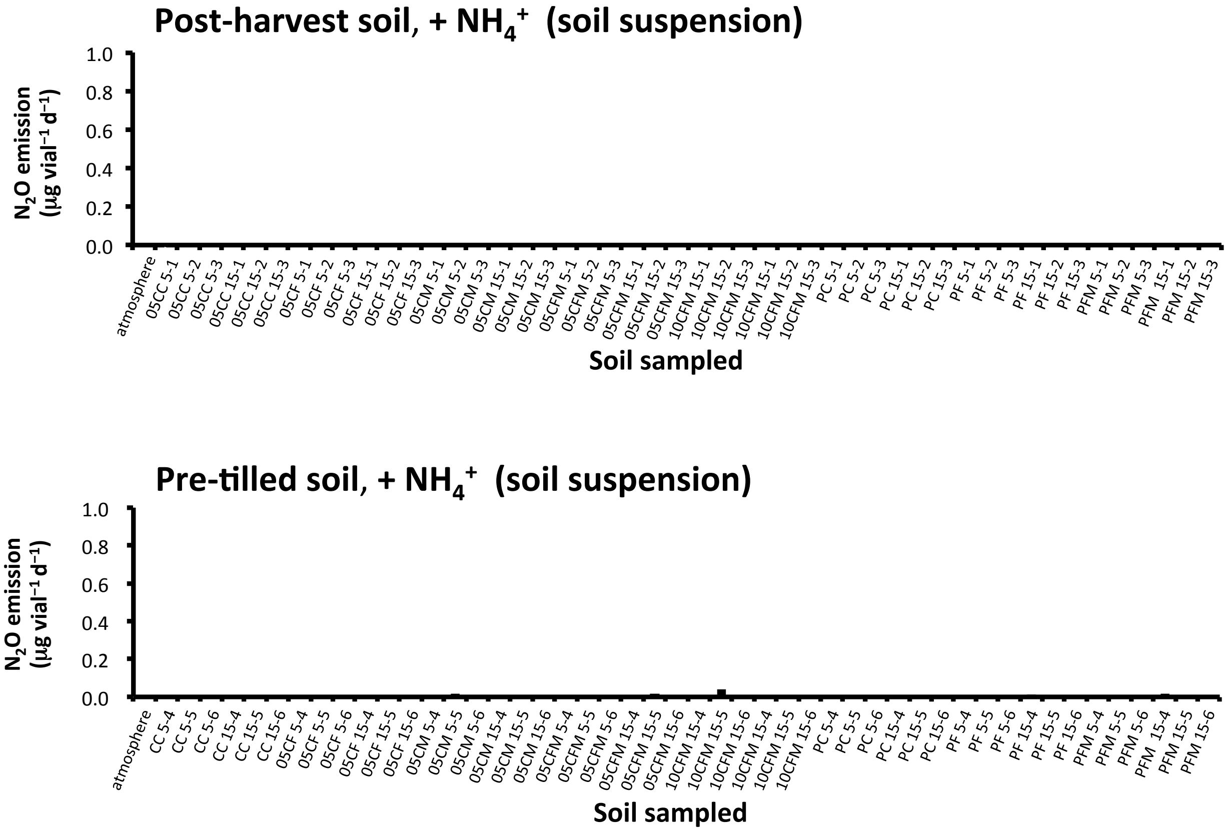

Figure S1. N_2O emission potential of soil microbial communities from corn farm Andisol in the culture medium supplemented with NH_4^+.

As a substrate for N_2O production, 500 mg NH_4^+ (as NH_4Cl) was used instead of 500 mg NO_3^- (as KNO_3). Other culturing conditions are the same as those in Fig. 1A and B.

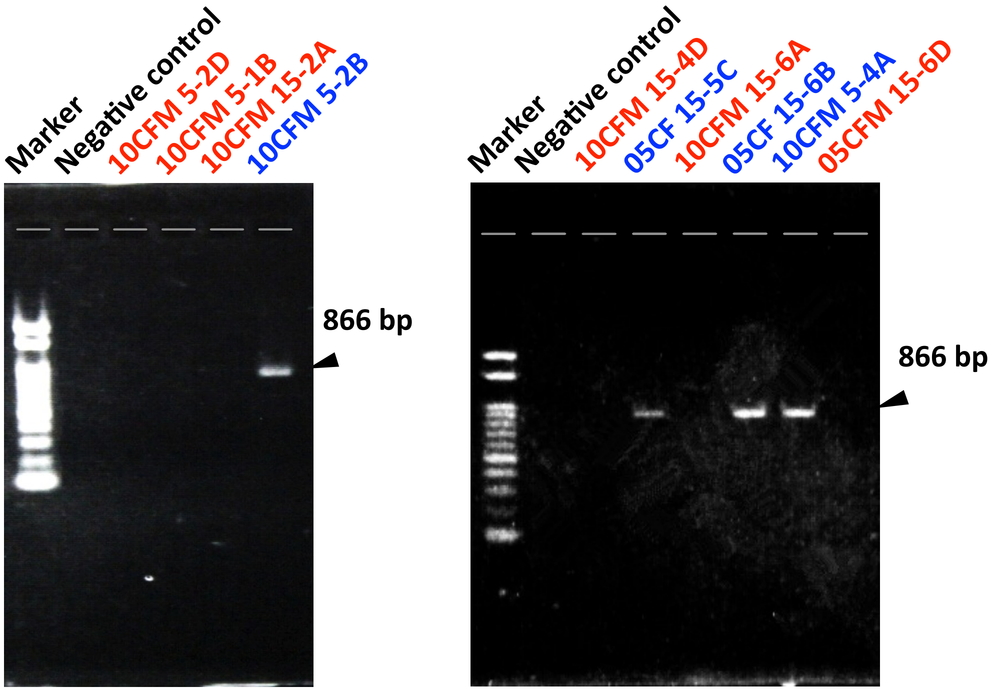

Bacterial isolates with blue characters (10CFM5-2B, 05CF15-5C, 05CF15-6B, and 10CFM5-4A) as *nosZ*-harboring denitrifiers

Figure S2. PCR assay for the detection of *nosZ* gene from the N_2O-emitting *Pseudomonas* isolated.

Sequences of partial *nosZ* gene obtained as PCR-amplicons using a primer set *nosZ-1111F* (5′-STA CAA CWC GGA RAA SG-3′) /*nosZ-1773R* (5′-ATR TCG ATC ARC TGB TCG TT-3′) for two isolates, *Pseudomonas* sp. 10CFM5-1B and 10CFM5-2B (left panel), and another set *nosZ-661F* (5′-CGG CTG GGG GCT GAC CAA-3′)/ *nosZ-1527R* (5′-CTG RCT GTC GAD GAA CAG-3′) for 10 pseudomonad from the post-harvest soils (left panel) and 6 isolates from the pre-tilled soils (right panel).

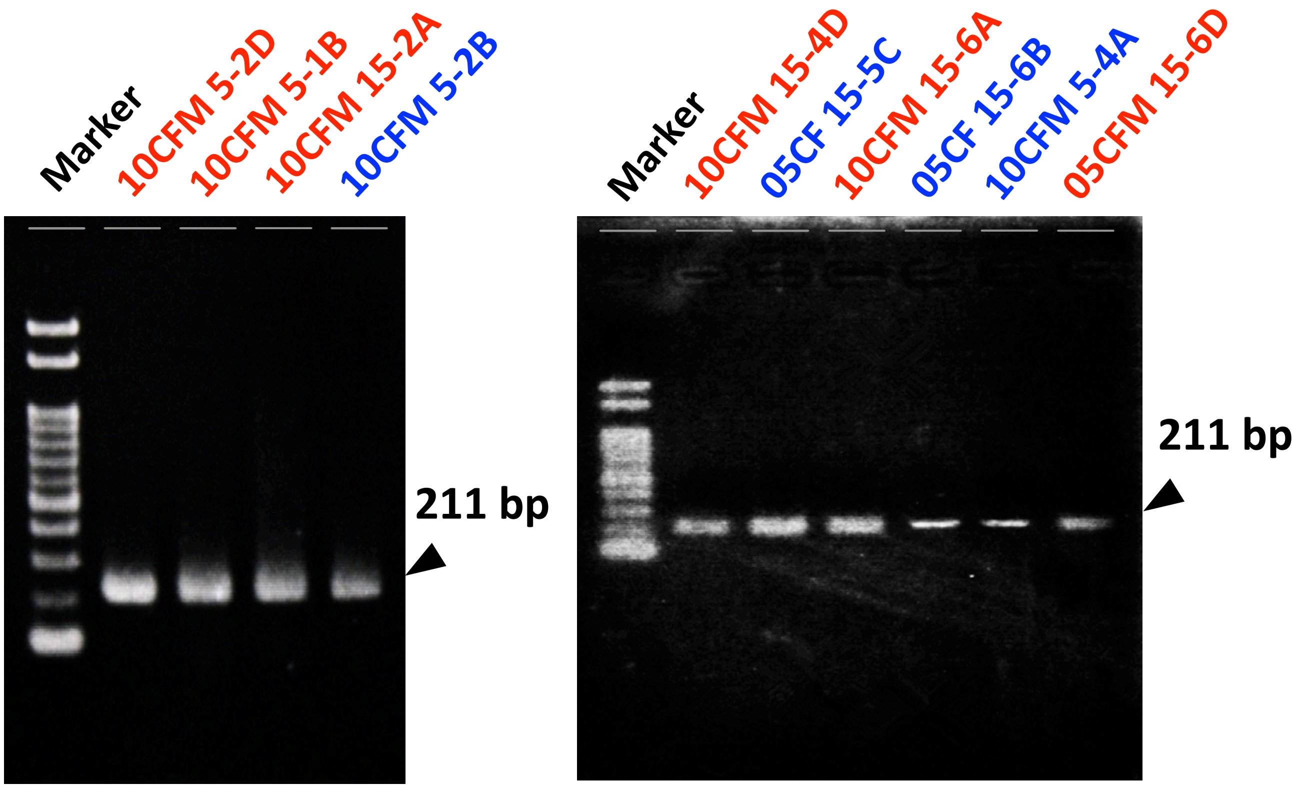

Figure S3. PCR assay for the detection of *narG* gene from the N_2O-emitting *Pseudomonas* bacteria.

Sequences of partial *narG* gene obtained as PCR-amplicons using a primer set *narG-Ps-2168F* (5′-TCG GGC AAG GGC CAT GAG TAC-3′) /*narG-Ps-2379R* (5′-TTT CGT ACC AGG TGG CGG TCG-3′). Left panel is an agarose gel plate for the denitrifiers from the post-harvest soils, and right panel is those from the pre-tilled soils.

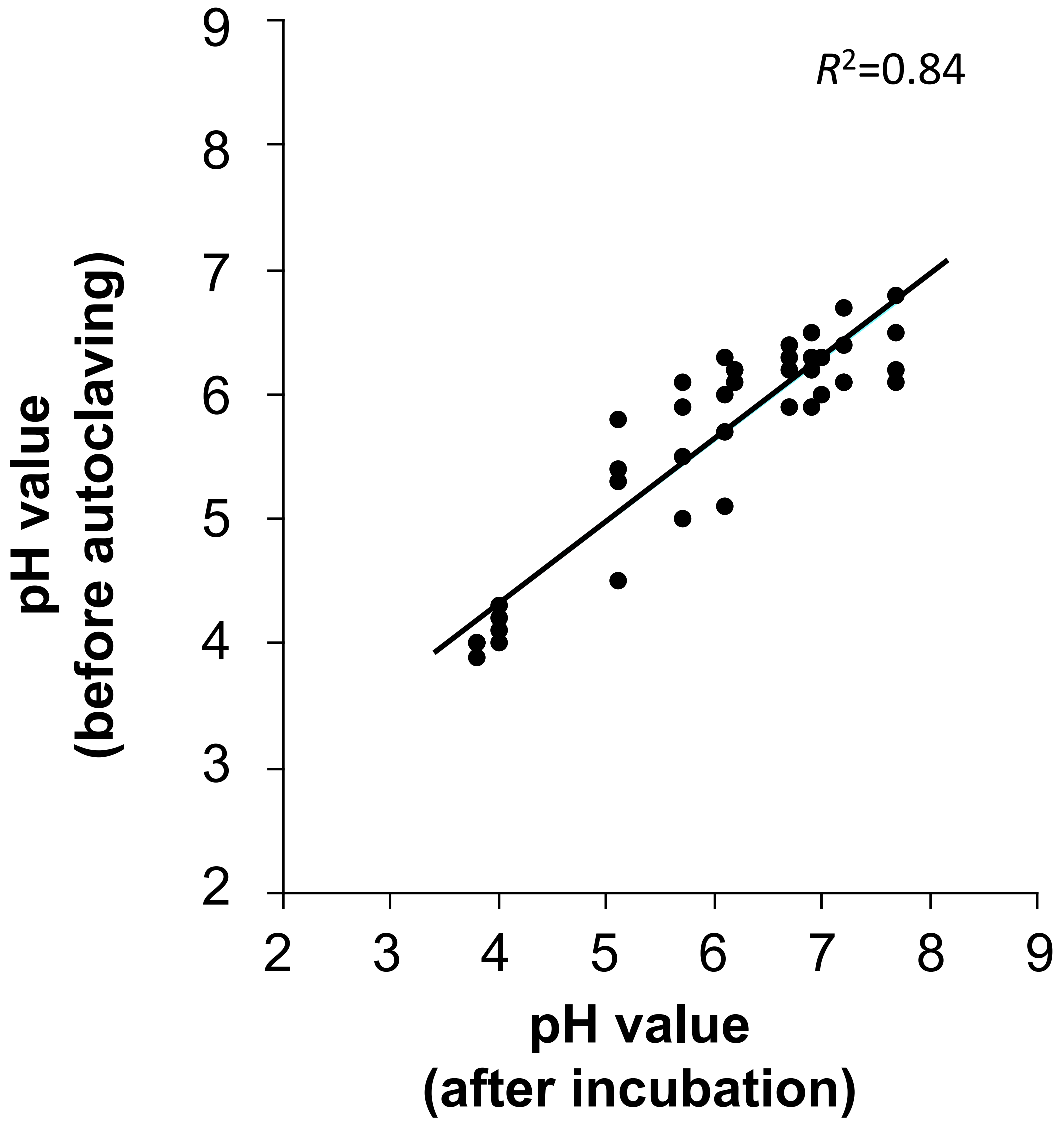

Figure S4. Values of medium pH before autoclaving and after autoclaved followed by 7-day-incubation.

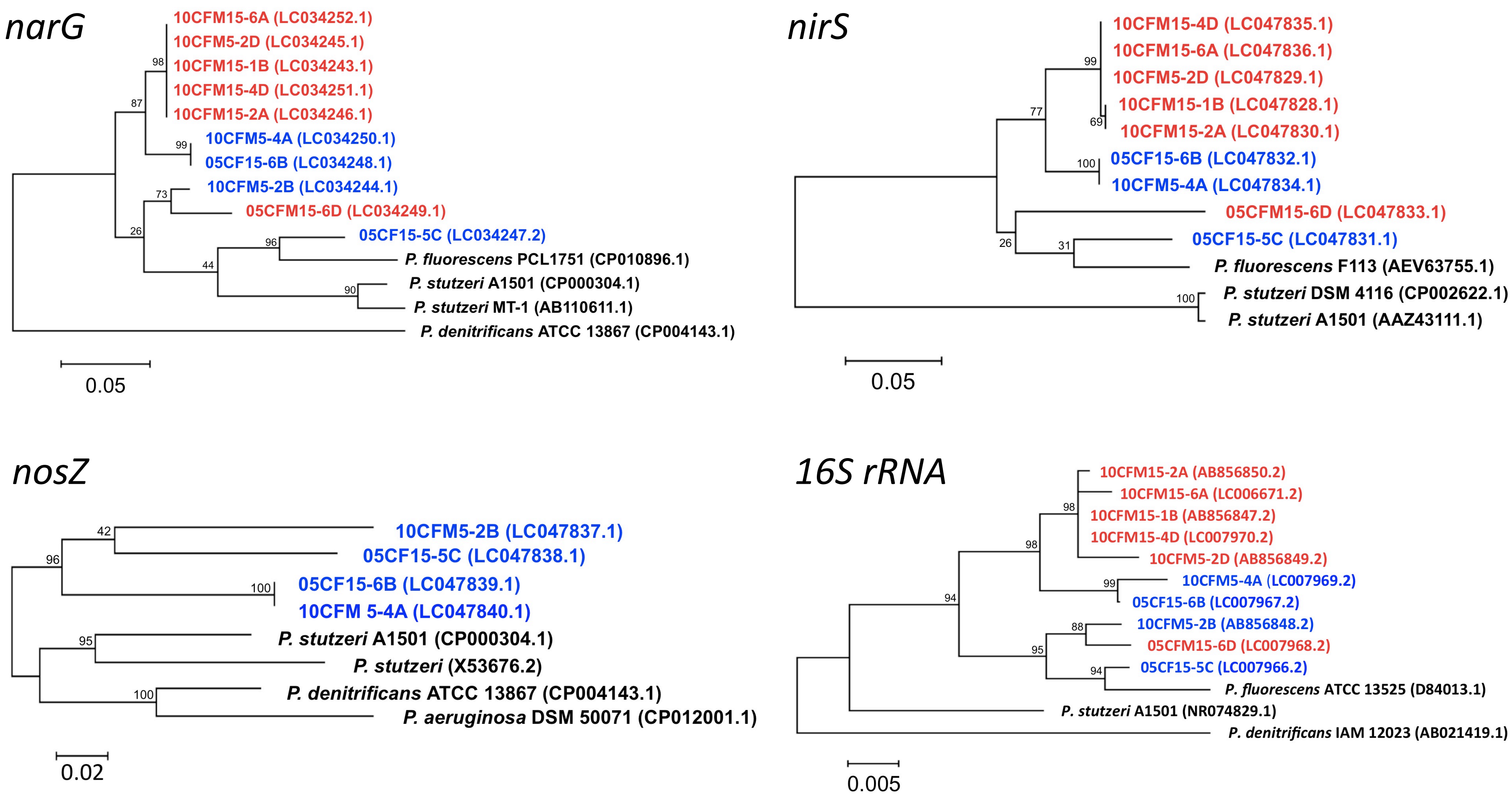

Figure S5. Phylogenetic analyses of the denitrification-associated genes from N_2O emitting pseudomonads using neighbor-joining tree.

The partial sequences of *narG*, *nirS*, and *nosZ* of the pseudomonads were subjected to phylogenetic analysis along with that of 16S rRNA gene, using MEGA 6.06 (Wineskin). Multiple alignments of the data collected from NCBI database were run using Clustal W and the phylogenetic tree was constructed using neighbor-joining methods with 1000 bootstrap replicates.

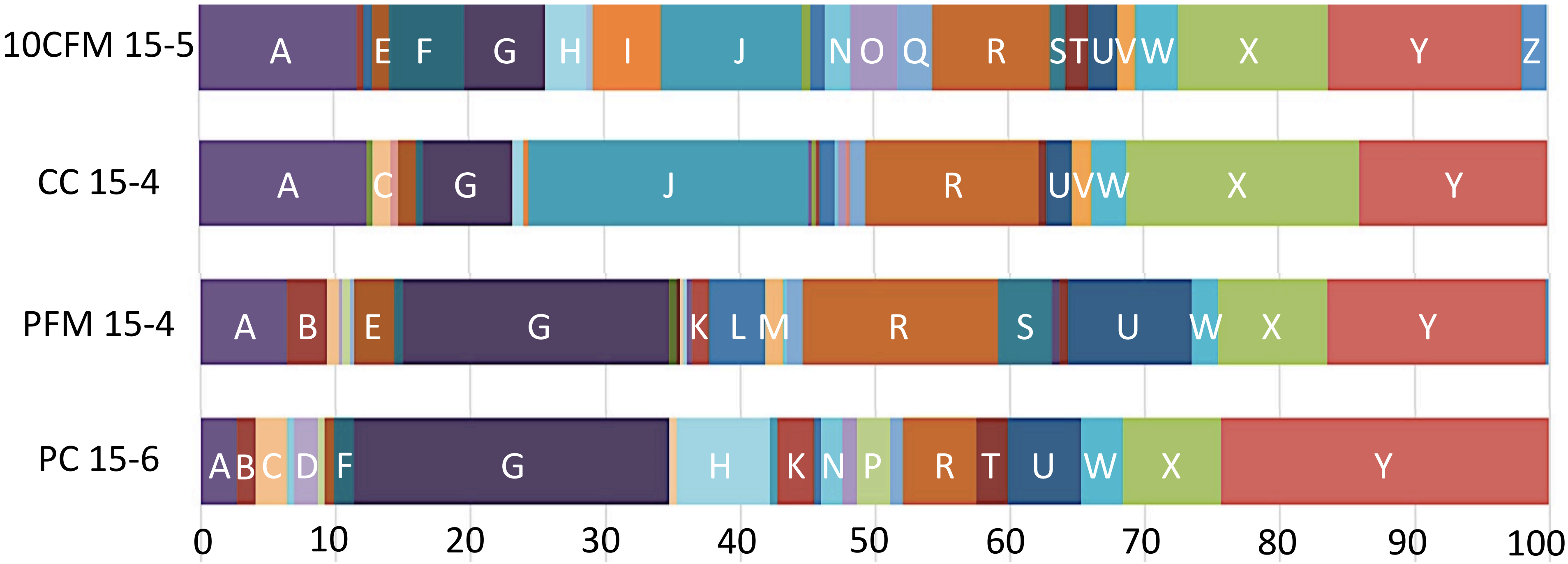

A, *Xanthomonadales*
B, unclassified *Dehalococcoidia*
C, *Thermoanaerobacter*
D, *Sphingomonadales*
E, *Rhodospirillales*
F, *Rhodocyclales*
G, *Rhizobiales*
H, *Nitrospirales*
I, *Myxococcales*
J, *Methylophilales*
K, *Gemmatimonadales*
L, *Gaiellales*
M, *Enterobacteriales*
N, *Desulfuromonadales*
O, *Desulfovibrionales*
P, *Dehalococcoidales*
Q, *Coriobacteriales*
R, *Clostridiales*
S, *Chromatiales*
T, *Caulobacteriales*
U, *Burkholderiales*
V, *Bacteroidales*
W, *Bacillales*
X, *Actinomycetales*
Y, *Acidobacteriales*
Z, *Acidimicrobiales*

Figure S6. Bacterial community structures of fertilized and unfertilized corm farm and pasture soils by 16S metagenomic analysis

Eubacterial community structure in each soil was shown by a taxon at the order level. 10CFM 15-5 is the soil from fertilized and manure-input corn farm, while CC 15-4 is that unfertilized corn farm soil without fertilization. PFM 15-4 and PC 15-6 are the soils from fertilized and unfertilized pasture respectively.
